# Reply to: Measurement of intracranial pressure and short-term outcomes of patients with traumatic brain injury: a propensity-matched analysis

**DOI:** 10.5935/0103-507X.20160037

**Published:** 2016

**Authors:** Cesar Biselli Ferreira, Luiz Marcelo Malbouisson, Estevão Bassi, Fernando Godinho Zampieri

**Affiliations:** 1 Trauma Intensive Care Unit, Hospital das Clínicas, Faculdade de Medicina, Universidade de São Paulo - São Paulo (SP), Brazil.; 2 Intensive Care Unit, Hospital Alemão Oswaldo Cruz - São Paulo (SP), Brazil.; 3 Intensive Care Unit, Discipline of Emergency Medicine, Hospital das Clínicas, Faculdade de Medicina, Universidade de São Paulo - São Paulo (SP), Brazil.

"Not everything that can be counted counts.

Not everything that counts can be counted".

Albert Einstein

We would like to thank you for the comments that have enriched this discussion. Although our manuscript fails to examine the data on the treatment of patients using intracranial pressure (ICP) monitoring, as Prof. Biestro emphasizes in his editorial,^(1)^ the striking datum might be the low overall mortality rate (< 16%), especially in a population in which < 10% of individuals are invasively monitored.^(2)^ Such data, and those from other authors,^(3)^ generate interest in the research on less invasive methods for managing patients with traumatic brain injury (TBI).

We have no doubt about the importance of the ICP monitor for the physiological and prognostic understanding of those patients. However, as Dr. Godoy emphasizes in his letter, many doubts exist regarding how to interpret ICP data and intervene in patient care. Several current interventions are prone to serious side effects and have questionable efficacy (for example, the use of barbiturates,^(4,5)^ hypothermia^(6)^ and a craniectomy.^(7)^ Thus, the possible benefit from more aggressive control of ICP using invasive monitors could be mitigated by the detriments associated with these interventions. For example, this issue becomes important in centers with high rates of infection because both hypothermia and the use of barbiturates are associated with increased risk of sepsis.^(4,8)^ Tsiolkovsky^(9)^ would most likely agree that the evidence that aggressive interventions for ICP handling may be harmful is not negligible and may not be ignored.

The above considerations explain another key caveat of our study (and others before us): what do patients with severe TBI die from? In other equally dramatic clinical situations, including acute respiratory distress syndrome, a minority of patients die from the dysfunction caused by the affected organ. Thus, how many patients with severe TBI indeed die from untreatable intracranial hypertension? Additionally, how many have worse outcomes due to complications resulting from the treatment? Early performance of a decompressive craniectomy resulted in improved intracranial hypertension control in the Decompressive Craniectomy in Diffuse Traumatic Brain Injury (DECRA) trial. However, the aggressive intervention was associated with worse 6-month outcomes.^(7)^

For all of these reasons, although monitoring using tomographic and sonographic methods is not dynamic, it may avoid overtreatment until we better understand how to intervene in the care of patients using ICP or multimodal monitoring.

Sample size calculation would make sense for a controlled, prospective study but not for a retrospective analysis.

We believe that the data supporting ICP control using invasive monitors are not sufficiently consistent to be conclusive. All difficulties mentioned by Dr. Godoy hinder a single and definitive randomized study on the subject; thus, observational studies may be extremely valuable, when contextualized, to assist in TBI patient care.

Cesar Biselli Ferreira and Luiz Marcelo Malbouisson

Trauma Intensive Care Unit, Hospital das Clínicas, Faculdade de Medicina, Universidade de São Paulo - São Paulo (SP), Brazil.

Estevão Bassi

Trauma Intensive Care Unit, Hospital das Clínicas, Faculdade de Medicina, Universidade de São Paulo - São Paulo (SP), Brazil; Intensive Care Unit, Hospital Alemão Oswaldo Cruz - São Paulo (SP), Brazil.

Fernando Godinho Zampieri

Intensive Care Unit, Hospital Alemão Oswaldo Cruz - São Paulo (SP), Brazil; Intensive Care Unit, Discipline of Emergency Medicine, Hospital das Clínicas, Faculdade de Medicina, Universidade de São Paulo - São Paulo (SP), Brazil.

## References

[1] Biestro A (2015). Intracranial pressure monitoring in the torture
chambre. Rev Bras Ter Intensiva.

[2] Ferreira CB, Bassi E, Lucena L, Carreta H, Miranda LC, Tierno PF (2015). Measurement of intracranial pressure and short-term outcomes of
patients with traumatic brain injury: a propensity-matched
analysis. Rev Bras Ter Intensiva.

[3] Cremer OL, van Dijk GW, van Wensen E, Brekelmans GJ, Moons KG, Leenen LP (2005). Effect of intracranial pressure monitoring and targeted intensive
care on functional outcome after severe head injury. Crit Care Med.

[4] Stover JF, Stocker R (1998). Barbiturate coma may promote reversible bone marrow suppression
in patients with severe isolated traumatic brain injury. Eur J Clin Pharmacol.

[5] Schalén W, Messeter K, Nordström CH (1992). Complications and side effects during thiopentone therapy in
patients with severe head injuries. Acta Anaesthesiol Scand.

[6] Andrews PJ, Sinclair HL, Rodriguez A, Harris BA, Battison CG, Rhodes JK, Murray GD, Eurotherm3235 Trial Collaborators (2015). Hypothermia for Intracranial hypertension after traumatic brain
injury. N Engl J Med.

[7] Cooper DJ, Rosenfeld JV, Murray L, Arabi YM, Davies AR, D'Urso P, Kossmann T, Ponsford J, Seppelt I, Reilly P, Wolfe R, DECRA Trial Investigators, Australian and New Zealand Intensive Care Society Clinical Trials
Group (2011). Decompressive craniectomy in diffuse traumatic brain
injury. N Engl J Med.

[8] Geurts M, Macleod MR, Kollmar R, Kremer PH, van der Worp HB (2014). Therapeutic hypothermia and the risk of infection: a systematic
review and meta-analysis. Crit Care Med.

[9] Konstantín Tsiolkovski.

